# Src-mediated tyrosine phosphorylation of Protein Kinase D2 at focal adhesions regulates cell adhesion

**DOI:** 10.1038/s41598-017-10210-7

**Published:** 2017-08-25

**Authors:** Nisha Durand, Ligia I. Bastea, Heike Döppler, Tim Eiseler, Peter Storz

**Affiliations:** 1 Department of Cancer Biology, Mayo Clinic, Jacksonville, Florida 32224 USA; 2 Department of Internal Medicine I, Ulm University, D-89081 Ulm, Germany

## Abstract

Dependent on their cellular localization, Protein Kinase D (PKD) enzymes regulate different processes including Golgi transport, cell signaling and response to oxidative stress. The localization of PKD within cells is mediated by interaction with different lipid or protein binding partners. With the example of PKD2, we here show that phosphorylation events can also contribute to localization of subcellular pools of this kinase. Specifically, in the present study, we show that tyrosine phosphorylation of PKD2 at residue Y87 defines its localization to the focal adhesions and leads to activation. This phosphorylation occurs downstream of RhoA signaling and is mediated via Src. Moreover, mutation of this residue blocks PKD2’s interaction with Focal Adhesion Kinase (FAK). The presence and regulation of PKD2 at focal adhesions identifies a novel function for this kinase as a modulator of cell adhesion and migration.

## Introduction

Protein kinase D 2 (PKD2), along with PKD1 and PKD3 constitute a family of serine/threonine kinases implicated in a wide variety of biological processes such as epithelial to mesenchymal transition, cell migration, proliferation, survival and angiogenesis^1, 2^. PKDs often have redundant functions, but recently, cellular responses where individual isoforms have unique targets were described. For example, PKD1 expression has been shown to block breast cancer cell migration and invasion *in vitro* and *in vivo* and to block matrix-metalloproteinase (MMP) expression^3–5^. In contrast to this, the two other isoforms promote these processes^6, 7^, and unlike PKD1, PKD2 induces cell invasion by regulating MMP expression and secretion^8, 9^. In addition to its effects on cell motility, PKD2 also has been implicated in enhancing tumor cell proliferation and tumor growth^10, 11^. Beyond its functions in cancer, PKD2, unlike the two other isoforms, also has been shown to have a critical role in T-cell antigen receptor signaling in mature T-cells; and in PKD2 deficient mice, loss of PKD2 is associated with enlarged lymph nodes and spleen^12, 13^.

In the absence of stimulation, PKD2 is mostly resident in the cytoplasm, but in response to receptor-mediated activation can translocate to the plasma membrane^14^. Moreover, its localization to other cellular sites such as the Golgi has been reported^8^. To date, there are no reports linking PKD2 to focal adhesion (FA) function, except one study indirectly showing that PKD (as detected with a pan-antibody that picks up PKD1 and PKD2), as well as cortactin and FA-localized paxillin can be isolated from invadopodia of breast cancer cells^15^.

In the present study, we show that tyrosine-phosphorylated PKD2 is localized at the focal adhesions. FAs are integrin-based macromolecular structures that link the actin cytoskeletal network within cells to matrix components^16^. FAs undergo constant flux, and their formation and dissolution is indispensable during cell adhesion and migration^16^. The FA complex is formed by a multitude of proteins, including adapters such as p130Cas and Paxillin, and the kinases focal adhesion kinase (FAK) and non-receptor tyrosine kinase Src^17^. FAK can autophosphorylate at Y397, which leads to binding and activation of Src. After activation, Src then phosphorylates a variety of FA proteins including FAK and p130Cas^18, 19^. The pivotal role of Src in the remodeling processes that contribute to the dynamic nature of FAs becomes evident after its inhibition, which results in a loss of integrin-mediated adhesions^20^ and FA turnover during cell migration^21^.

In this report, we define PKD2 as a new target for Src at the focal adhesions. We describe phosphorylation at Y87 as a defining characteristic of PKD2 localization to the focal adhesions. We further show that RhoA functions upstream of Src in mediating this phosphorylation, and that inhibition of Y87 phosphorylation by RhoA/Src impairs cell adhesion and migration.

## Results

### Y87-phosphorylated PKD2 can be detected at the focal adhesions

**Figure 1:**
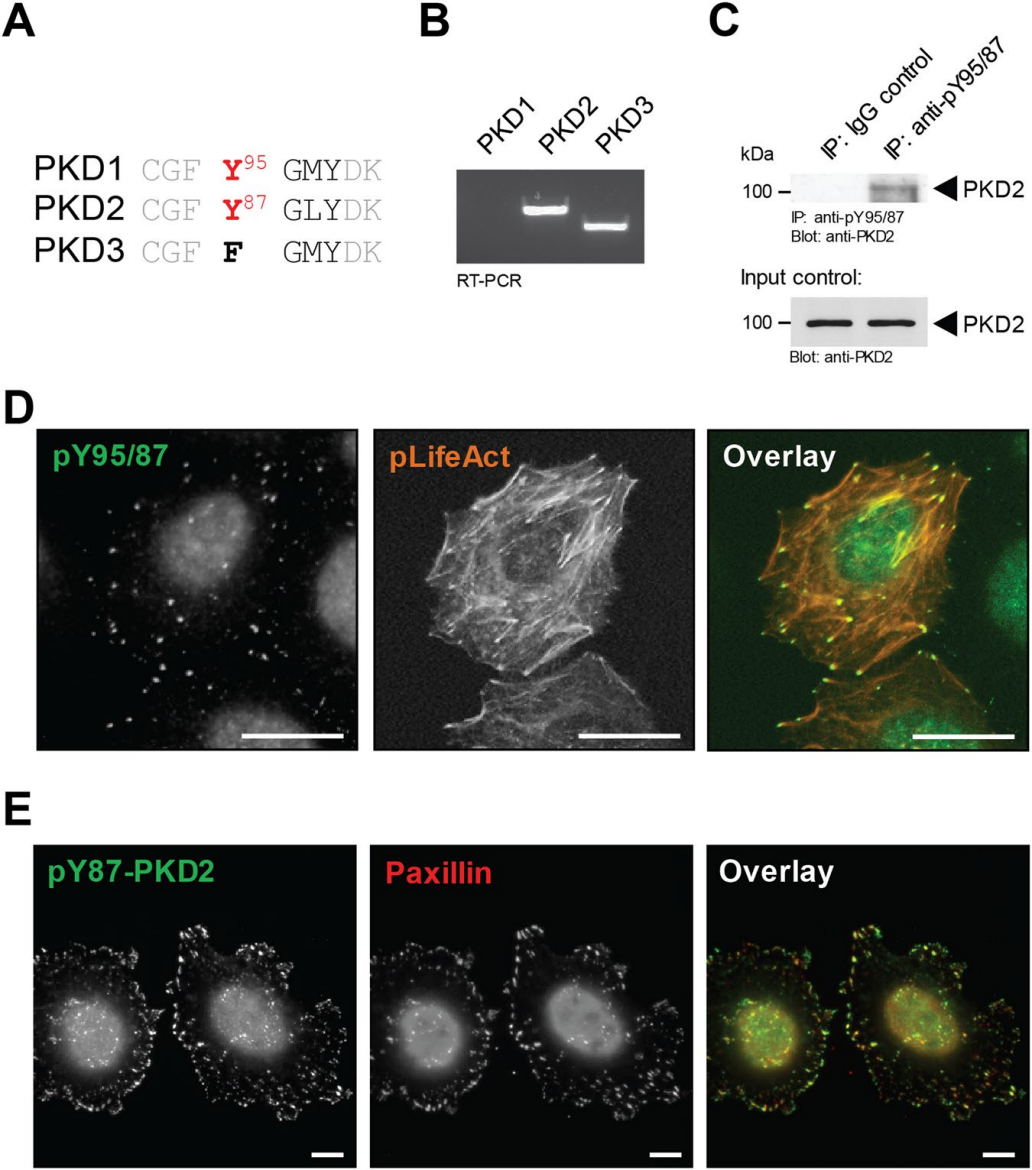
Y87-phosphorylated PKD2 can be detected at the focal adhesions. (**A)** Comparison of the consensus motif required for Src phosphorylation in the 3 PKD isoforms. (**B)** RT-PCR analysis of PKD1, PKD2 and PKD3 expression in HeLa cells. (**C)** HeLa cells (3.5 × 10^6^ cells per 10 cm plate) were subjected to immunoprecipitation analysis using the anti-pY95/87 antibody, and then probed for PKD2. Control Western blots of lysates were evaluated for PKD2 expression (input). Uncropped blots are shown in Supplemental Figure S7. (**D**) HeLa cells in ibidi channel μ-slides were transfected with RFP-LifeAct for visualization of F-actin. Cells were fixed and the localization of endogenous pY87-PKD2 was determined by immunofluorescence analysis (secondary antibody used: Alexa Fluor 488). Scale bars indicate 50 μm. (**E)** HeLa cells were seeded in ibidi channel μ-slides to determine the localization of endogenous pY87-PKD2 and paxillin (secondary antibodies used: Alexa Fluor 488, Alexa Fluor 568). Scale bars indicate 10 μm. All experiments have been performed at least three times with similar results.

Of the three PKD isoforms, only PKD1 and PKD2 contain a previously-described pY-G-M/L-Y motif (Fig. 1A), which in PKD1 is phosphorylated downstream of Src^22^. In order to study the role of tyrosine phosphorylation of PKD2 at this residue, HeLa cells, as well as NMuMG and MDA-MB-231 cells were deemed as an ideal systems, because they express only PKD2 and PKD3 (Fig. 1B, Supplemental Figure S1A, and ref. 4), and therefore all data with our phosphospecific antibody for this residue (Y95 in PKD1 and Y87 in PKD2) could be attributed to PKD2 without any confounding effects from PKD1. Immunoprecipitation using the anti-pY95/Y87 antibody and detection for PKD2 indicated that this antibody indeed recognizes PKD2 phosphorylated at Y87 (Fig. 1C), while probing for PKD3 produced the expected negative result (Supplemental Figure S1B). We next determined at which cellular localization this phosphorylation event may occur. In immunofluorescence analysis of HeLa cells the pY95/Y87 antibody produced a punctate staining pattern in which F-actin filaments terminated (Fig. 1D). In order to confirm that these are indeed FAs, we performed co-immunofluorescence staining of cells with pY95/87antibody and the *bona fide* FA marker paxillin, and found that both co-localize (Fig. 1E). Similar co-localization was observed with NMuMG and MDA-MB-231 cells (Supplemental Figure S1C), indicating that this is not a cell type specific effect.

In order to show that a cellular pool of PKD2 can localize to the focal adhesions, we utilized a co-immunoprecipitation approach, and found that paxillin can be detected in PKD2 immunoprecipitates (Supplemental Figure S2A). However, the detection of PKD2 at the FAs via immunofluorescence using PKD2 antibodies proved to be challenging. This was due to antibodies available as well as the fact that the majority of this kinase is localized in these cells in the cytosolic, nuclear and perinuclear regions. An apical to basal analysis of PKD2 expression indicated this ubiquitous localization, and only a very small fraction of PKD2 can be detected at the cellular edge and potential focal adhesions (Supplemental Figure S2B).

**Figure 2:**
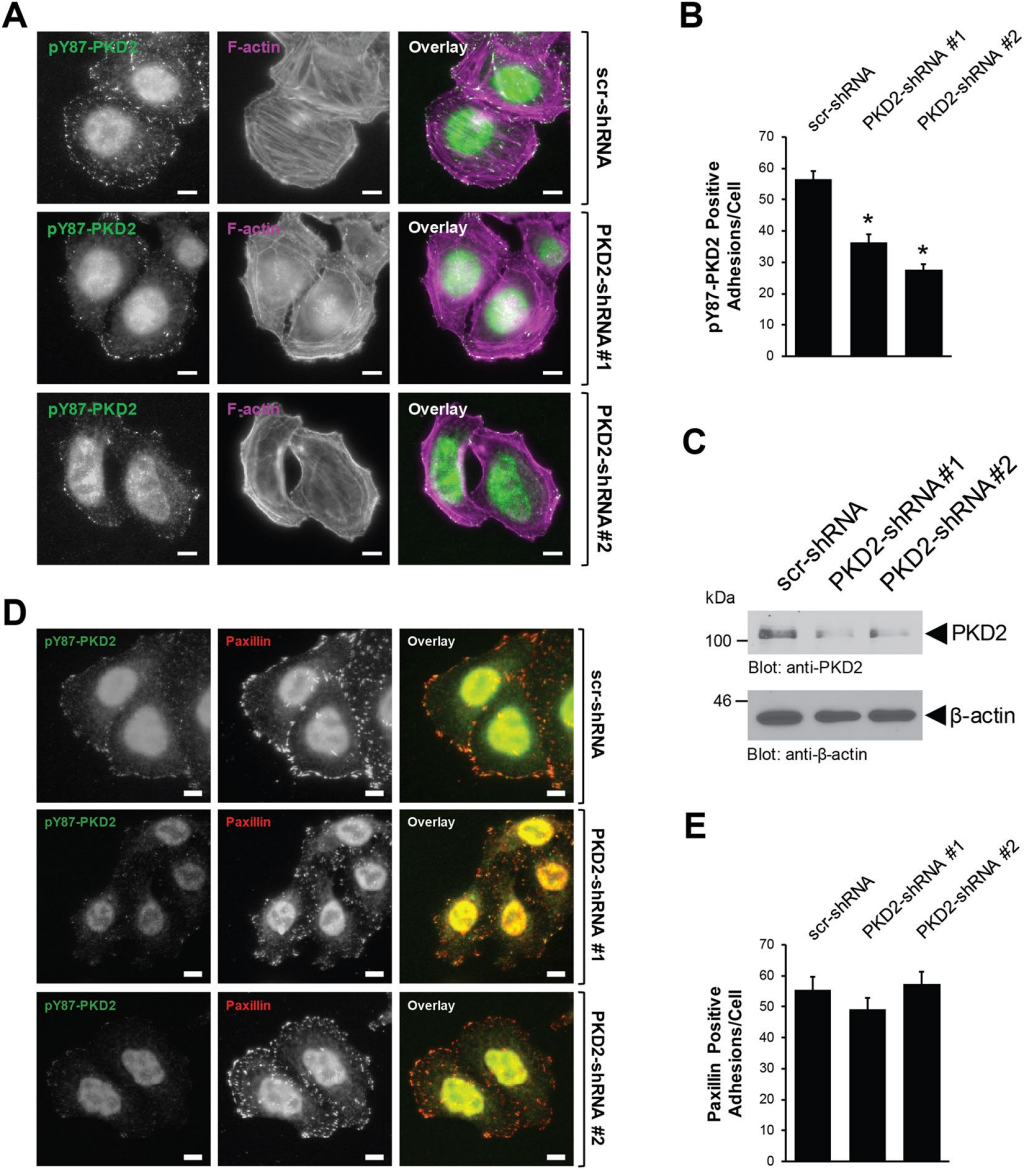
The anti-pY87 signal at the focal adhesions corresponds to PKD2. (**A)** HeLa cells were infected with lentivirus expressing non-target shRNA (scr-shRNA), or two independent shRNA sequences targeting PKD2 (PKD2-shRNA#1, PKD2-shRNA#2). After 48 hours, cells (6000/channel) were reseeded in ibidi channel μ-slides. Cells were subjected to immunofluorescence analysis to determine the localization of endogenous pY87-PKD2 (secondary antibody used: Alexa Fluor 488) and F-actin (phalloidin). Scale bar indicates 10 μm. (**B)** Shows quantitation for the number of pY87-PKD2-positive adhesions per cell with scr-shRNA (n = 73 cells), PKD2-shRNA#1 (n = 63 cells) and PKD2-shRNA#2 (n = 64 cells). Error bars represent standard error of the mean (SEM). *Indicates statistical significance (*p* < 0.05) as compared to cells expressing scr-shRNA. (**C)** Cell lysates from A were evaluated by Western blotting for the expression of PKD2 and β-actin. All experiments have been performed at least three times with similar results. Uncropped blots are shown in Supplemental Figure S7. (**D)** HeLa cells were infected and reseeded in ibidi channel μ-slides, as described in (**A)** Cells were subjected to immunofluorescence analysis to determine the localization of endogenous pY87-PKD2 and paxillin. Scale bar indicates 10 μm. (**E)** Shows quantitation for the number of paxillin-positive adhesions per cell with scr-shRNA (n = 40 cells), PKD2-shRNA#1 (n = 40 cells) and PKD2-shRNA#2 (n = 40 cells). Error bars represent standard error of the mean (SEM). *Indicates statistical significance (*p* < 0.05) as compared to cells expressing scr-shRNA.

In order to show that pY87-PKD2 is indeed present at FAs we performed a reverse-genetic analysis using two independent shRNAs targeting PKD2. The knockdown of PKD2 led to a significant decrease in the number of pY87-PKD2 adhesions in cells indicating that the signal observed with the phosphosite-specific antibody is indeed due to Y87-phosphorylayed PKD2 (Fig. 2A). Quantification analysis of the pY87-PKD2 positive adhesions across the three experimental conditions (Fig. 2B) revealed that compared to control cells, the loss of PKD2 was associated with the loss of pY87 positive adhesions; in one instance, about 40% (PKD2-shRNA#1) and in the other, about 50% (PKD2-shRNA#2). This somewhat reflected the grade of knockdown detected by Western blot (Fig. 2C). While pY87-PKD2 decreased at the focal adhesions, when PKD2 was knocked down, the numbers of paxillin-positive FA remained unchanged under all conditions (Fig. 2D, E). This indicates that the knockdown of PKD2 does not affect FA formation. Loss of PKD2 was also associated with a moderate but significant decrease in cell spreading as measured by an approximately 20% reduction in cell area covered (Supplemental Figure S3A, B).

### Y87-phosphorylation of PKD2 is mediated by active Src

**Figure 3:**
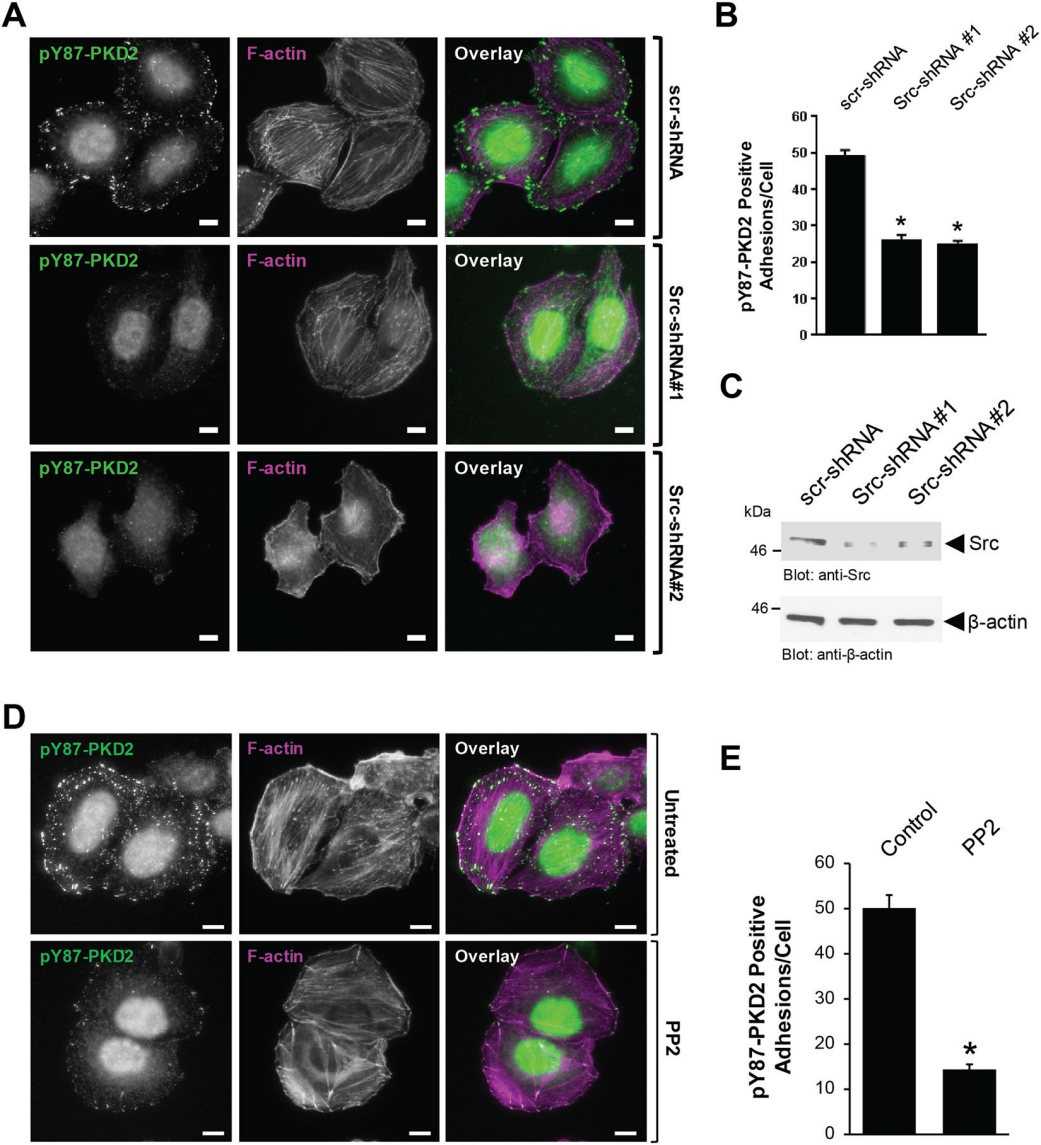
Y87-PKD2 at the focal adhesions is regulated by Src. (**A)** HeLa cells were infected with lentivirus expressing non-target shRNA (scr-shRNA), or two independent shRNA sequences targeting Src (Src-shRNA#1, Src-shRNA#2). After 48 hours, cells (6000/channel) were reseeded in ibidi channel μ-slides. Cells were subjected to immunofluorescence analysis to determine the localization of endogenous pY87-PKD2 (secondary antibody used: Alexa Fluor 488) and F-actin (phalloidin). Scale bar indicates 10 μm. (**B)** Shows quantitation for the number of Y87-PKD2-positive adhesions per cell with scr-shRNA (n = 53 cells), Src-shRNA#1 (n = 51 cells) and Src-shRNA#2 (n = 53 cells). Error bars represent standard error of the mean (SEM). *Indicates statistical significance (*p* < 0.05) as compared to cells expressing scr-shRNA. (**D)** Cell lysates from A were evaluated by Western blotting for the expression of Src and β-actin. Uncropped blots are shown in Supplemental Figure S7. (**D)** HeLa cells (6000/channel) were seeded in ibidi channel μ-slides with 2.5 μM PP2 or left untreated. After 24 hours, cells were subjected to immunofluorescence analysis to determine the localization of endogenous pY87-PKD2 (secondary antibody used: Alexa Fluor 488) and F-actin (phalloidin). Scale bar indicates 10 μm. (**E)** Shows quantitation for the number of pY87-PKD2-positive adhesions per cell with control (n = 80 cells) and PP2-treated cells (n = 77 cells). Error bars represent standard error of the mean (SEM). *Indicates statistical significance (*p* < 0.05) as compared to control cells. All experiments have been performed at least three times with similar results.

We previously have shown that PKD1 can be phosphorylated at Y95 in response to Src^22^. Therefore, we next determined if PKD2 phosphorylation at the Y87 site present at the focal adhesions is phosphorylated downstream of Src. In HeLa cells grown in channel slides, active Src, as measured by its phosphorylation at Y418, can be detected at the focal adhesions (Supplemental Figure S4), and a knockdown of Src led to a significant decrease in the number of pY87-PKD2 adhesions in cells (Fig. 3A). Quantification analysis of the pY87-PKD2 positive adhesions (Fig. 3B) revealed that compared to control cells, a decrease in Src was associated with the loss of pY87 positive adhesions (about 50% for both Src-shRNAs), and reflected the grade of knockdown detected by Western blot (Fig. 3C). In addition, the inhibition of Src family kinases by PP2 significantly decreased the presence of pY87-PKD2 at focal adhesions (Fig. 3D). Quantification analysis revealed that there was a 70% reduction in pY87-positive adhesions in cells treated with PP2 in comparison to the untreated control cells (Fig. 3E).

**Figure 4:**
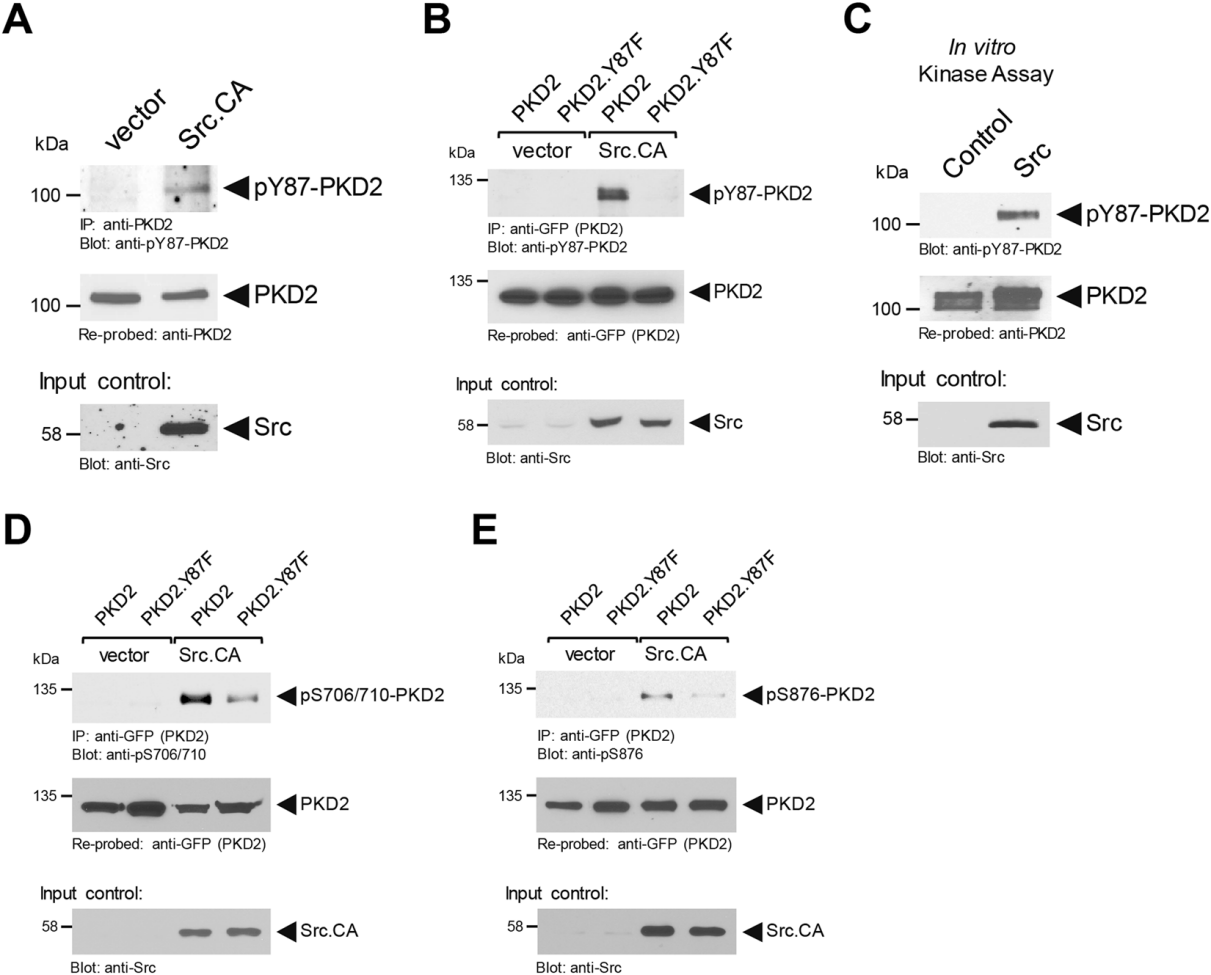
Y87-phosphorylation of PKD2 is mediated by active Src. (**A)** HeLa cells (2 × 10^6^ cells/10 cm plate) were transfected with vector or constitutively-active Src (Src.CA) as indicated. PKD2 was immunoprecipitated and analyzed using the pY87-PKD2 antibody. Samples were counterstained for total PKD2 and lysates were assessed by Western blot for Src expression (input control). (**C**) Src *in vitro* kinase assay using purified PKD2 as a substrate. After the kinase reaction samples were analyzed for Y87 phosphorylation using the pY87-PKD2 antibody. Samples were counterstained for total PKD2 and for Src (input controls). (**B**,**D**,**E**) HeLa cells (0.75 × 10^6^ cells/6-cm plate) were co-transfected with vector or constitutively-Src (Src.CA) and GFP-tagged versions of PKD2 or PKD2.Y87F as indicated. PKD2 was immunoprecipitated (anti-GFP) and assessed for Src-mediated phosphorylation using the pY87-PKD2 antibody (**B**), assessed for PKD2 activation loop phosphorylation using an anti-pS706/710 antibody (**D**), or for PKD2 autophosphorylation using an anti-pS876 antibody (**E**). Immunoprecipitates were re-probed with anti-GFP for expression control of PKD2 and PKD2.Y87F. The lysates were evaluated by Western blot for Src expression (input control). Uncropped blots for Fig. 4 are shown in Supplemental Figures S7, S8 and S9. All experiments have been performed at least three times with similar results.

We next assessed the ability of Src to induce the phosphorylation of PKD2. Therefore, we expressed constitutively-active Src in HeLa cells growing on plastic and probed for Y87 phosphorylation of endogenous PKD2. While in control transfected cells endogenous PKD2 was not phosphorylated, the presence of active Src induced phosphorylation at Y87 (Fig. 4A). To further prove the specificity of our antibody and to show that this Src-mediated phosphorylation of PKD2 occurs at Y87, we co-expressed PKD2 or a PKD2.Y87F mutant together with vector control or active Src (Fig. 4B). We then determined if Src can phosphorylate PKD2 directly. Therefore, we performed an *in vitro* kinase assay using purified PKD2 and Src, and found a direct phosphorylation at Y87 (Fig. 4C). For PKD1, phosphorylation at the equivalent tyrosine residue (Y95), leads to phosphorylation of its activation loop and subsequent activation^22^. Similarly, Src-mediated phosphorylation of PKD2 induced activation loop phosphorylation of PKD2 at S706/S710, as well as autophosphorylation at S876, both indicative for active PKD2, and this was significantly reduced in the PKD2.Y87F mutant (Figs. 4D, E). This indicates that Y87 phosphorylation of PKD2 by Src is implicated in activation of the kinase at the focal adhesions.

### Src acts downstream of RhoA to mediate Y87-phosphorylation of PKD2

**Figure 5:**
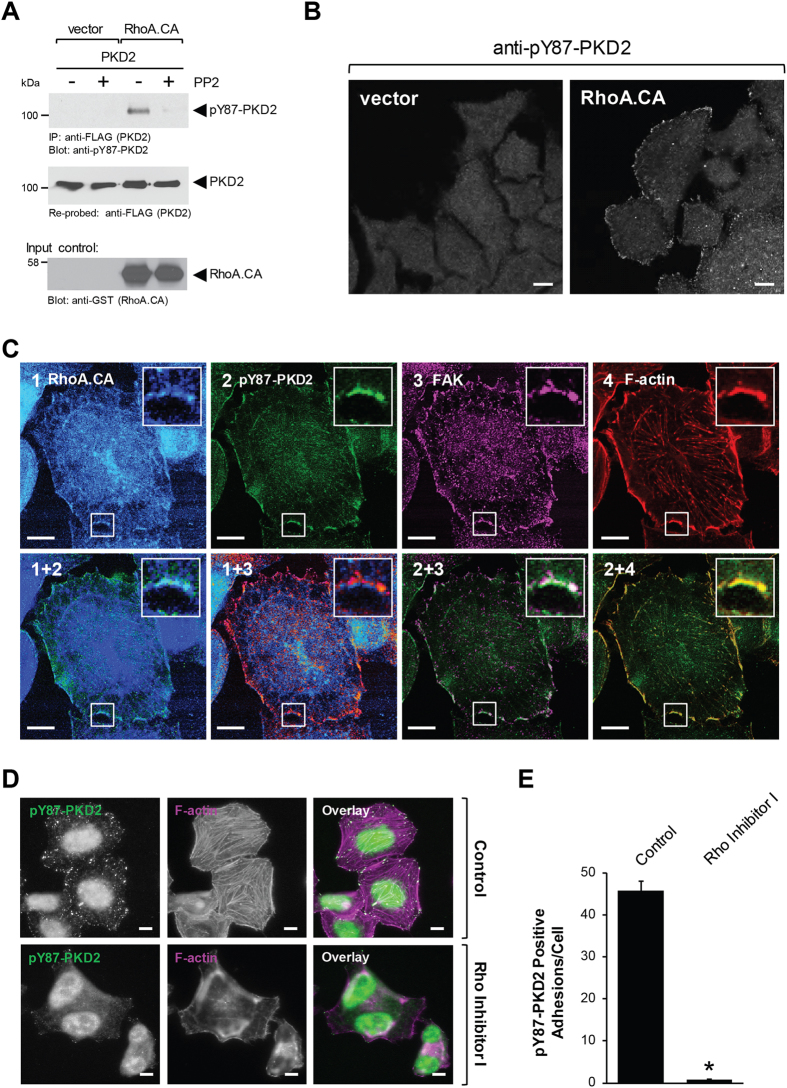
Src acts downstream of RhoA to mediate Y87-phosphorylation of PKD2. (**A**) HeLa cells (0.65 × 10^6^ cells/6 cm plate) were co-transfected with FLAG-PKD2 and vector or constitutively-active RhoA (RhoA.CA). Cells were stimulated with PP2 (2.5 μM, 16 hours) or DMSO, as indicated. PKD2 was immunoprecipitated (anti-FLAG) and samples were analyzed for Y87 phosphorylation using the anti-pY87-PKD2 antibody. Immunoprecipitates were re-probed for total PKD2 and cell lysates were evaluated by Western blot for RhoA.CA (anti-GST) expression. Uncropped blots are shown in Supplemental Figure S9. (**B**) HeLa cells, transfected with vector control or GST-tagged constitutively-active RhoA (RhoA.CA), were seeded on coverslips (70,000 cells/slip). 24 hours later, cells were analyzed by immunofluorescence for phosphorylation at Y87 (anti-pY95/87 and Alexa Flour 546). (**C)** HeLa cells, transfected with GST-tagged constitutively-active RhoA (RhoA.CA), were seeded on coverslips (70,000 cells/slip). 24 hours later they were analyzed by immunofluorescence for phosphorylation at Y87 (anti-pY95/87 labeled with Zenon Alexa Flour 488), for FAK (anti-FAK labeled with Zenon Alexa Flour 647) and for GST (anti-GST labeled with Zenon Alexa Flour 405). F-actin filaments were visualized using rhodamine-Phalloidin. (**D)** HeLa cells (6000/ channel) were seeded in ibidi channel μ-slides with 2 μg/ml Rho Inhibitor I, as indicated. After 16 hours, cells were subjected to immunofluorescence analysis to determine the localization of endogenous pY87-PKD2 (secondary antibody used: Alexa Fluor 488) and F-actin (phalloidin). Scale bar indicates 10 μm. (**E)** Shows quantitation for the number of pY87-PKD2-positive adhesions per cell with control (n = 65 cells) and Rho Inhibitor l-treated cells (n = 65 cells).Error bars represent standard error of the mean (SEM). *Indicates statistical significance (*p* < 0.05) as compared to control cells. All experiments have been performed at least three times with similar results.

PKD activity can be induced downstream of RhoA^23^, and according to several reports RhoA and Src can function in the same signaling pathway to regulate processes like cell migration and cell polarity^24–26^. Therefore, we explored if RhoA can lead to Src-mediated phosphorylation of PKD2. We found that expression of an active version of RhoA (RhoA.CA), but not Rac1 (Rac1.CA) or Cdc42 (Cdc42.CA), can induce phosphorylation of PKD2 at Y87 (Supplemental Figure S5). This was completely blocked when Src was inhibited with PP2, indicating that in this signaling cascade Src family kinases function downstream of RhoA (Fig. 5A).

Since cells, when seeded in channel slides, showed PKD2 phosphorylation at the FAs, suggesting a possible constitutive-signaling in the channel, in order to determine the upstream signaling leading to this event, we seeded cells on coverslips or in normal culture dishes. In cells transfected with RhoA.CA, Y87-phosphorylated PKD2 was detected at the focal adhesions (Fig. 5B), where it co-localized with RhoA and FAK and F-actin (Fig. 5C). Moreover, the treatment of cells growing in channel slides with Rho Inhibitor I led to a decrease of Y87 phosphorylation, along with drastic changes in cellular morphology and loss of actin stress fibers (Fig. 5D). Accordingly, when we quantified the pY87-PKD2 positive adhesions in cells, we found that Rho inhibition resulted in an over 90% decrease in the number of focal adhesions positive for pY87-PKD2 (Fig. 5E). Together, these results indicate that Y87 phosphorylation of PKD2 is mediated by a RhoA-Src pathway.

### Prevention of Y87-phosphorylation decreases cell adhesion and cell migration

**Figure 6:**
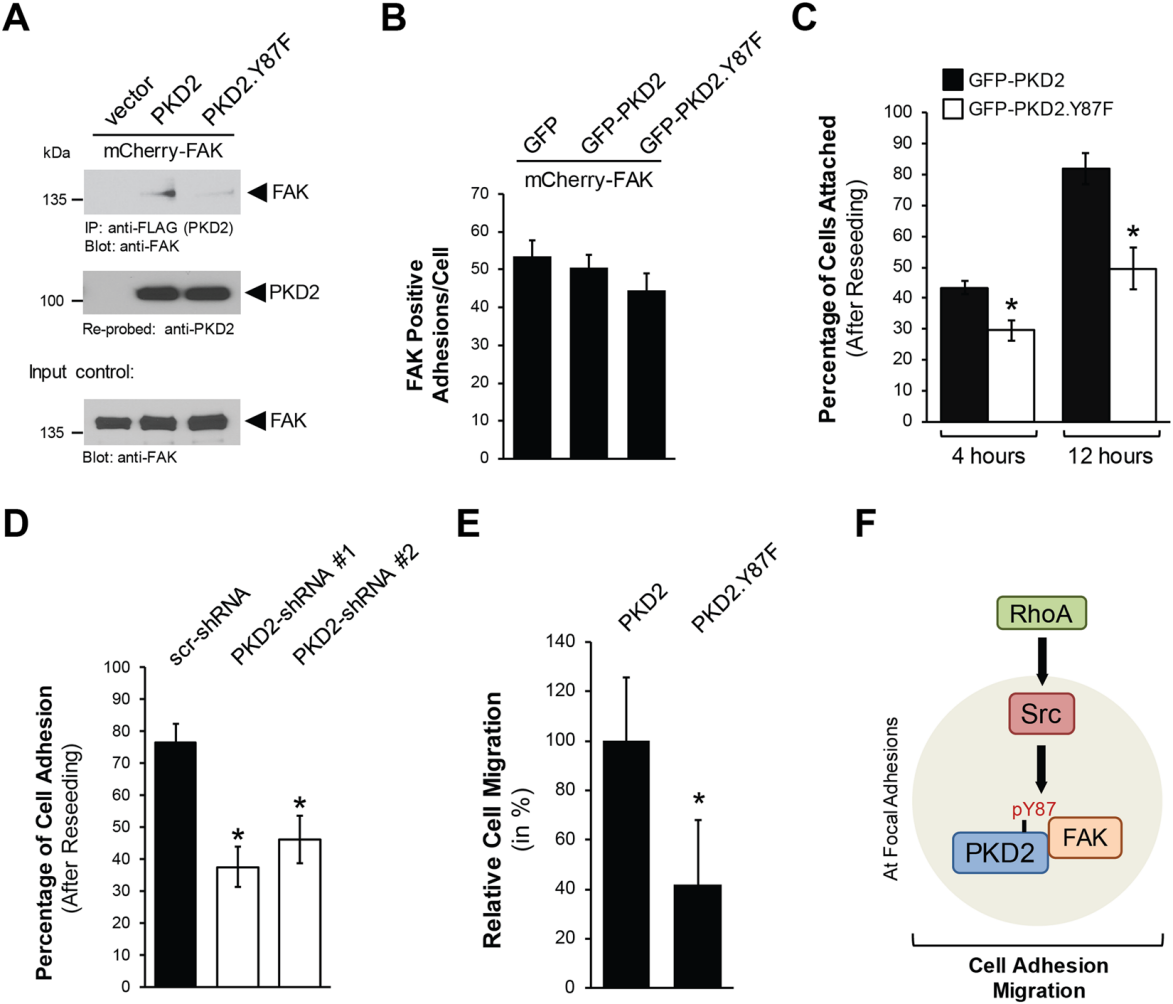
Prevention of Y87-phosphorylation decreases cell adhesion and cell migration. (**A)** Hek293T cells (0.5 × 10^6^ cells/well, 6 well plate) were co-transfected with vector control or FLAG-tagged versions of PKD2 or PKD2.Y87F together with mCherry-FAK. PKD2 was immunoprecipitated (anti-FLAG) and analyzed for co-immunoprecipitated FAK. Immunoprecipitates were then re-probed for PKD2. Control Western blot was probed for FAK expression. Uncropped blots are shown in Supplemental Figure S9. (**B)** HeLa cells were co-transfected with GFP or GFP-tagged versions of PKD2 or PKD2.Y87F together with mCherry-FAK and then re-plated on fibronectin-coated (2 μg/ml) glass coverslips at a density of 0.03 × 10^6^ cells per coverslip. Fluorescent images were acquired and numbers of FAK-positive adhesions were determined. Shown is a quantitation for the number of FAK-positive adhesions per double transfected cell, with GFP (n = 50 cells), GFP-PKD2 (n = 39 cells) and GFP-PKD2.Y87F (n = 46 cells). (**C**) HeLa cells were transfected with GFP-tagged versions of PKD2 or PKD2.Y87F and then re-plated on fibronectin-coated (10 μg/ml) glass coverslips at a density of 0.02 × 10^6^ cells per coverslip. Fluorescent images were acquired at the indicated times to determine the number of cells attached. Shown is a quantitation for the percentage of cells attached at 4 and 12 hours (images are shown in Supplemental Figure S6B). Error bars represent standard error of the mean (SEM). * indicates statistical significance (*p* < 0.05) as compared to wildtype control at the time point measured. (**D)** HeLa cells were infected with lentivirus expressing non-target shRNA (scr-shRNA), or two independent shRNA sequences targeting PKD2 (PKD2-shRNA#1, PKD2-shRNA#2). After 48 hours, cells were re-plated on fibronectin-coated (10 μg/ml) glass coverslips at a density of 0.02 × 10^6^ cells per coverslip. Fluorescent images were acquired to determine the number of cells attached. Shown is a quantitation for the percentage of cells attached at 8 hours. Error bars represent standard error of the mean (SEM). * indicates statistical significance (*p* < 0.05) as compared to wildtype control at the time point measured. (**E)** HeLa cells were transfected with GFP-tagged versions of PKD2 or PKD2.Y87F and then seeded on Transwell plates. After 16 hours, cells that had migrated to the lower surface of the filters were fixed in 4% paraformaldehyde and counted. Shown is the relative cell migration with PKD2 set as 100%. Error bars represent standard error of the mean (SEM). *Indicates statistical significance (*p* < 0.05) as compared to wildtype control at the time point measured. (**F)** Model of how PKD2 tyrosine phosphorylation at Y87 is regulated at the focal adhesions and functional consequences of this event on cell adhesion and migration.

We next determined the functional consequences of PKD2 phosphorylation via RhoA-Src. The mutation of PKD2 at Y87 prevented its interaction with FAK (Fig. 6A), but does not lead to a decrease in numbers of FAK-positive focal adhesions within cells (Fig. 6B). The recruitment of proteins to the FAs is required for many processes including cell adhesion to the ECM and cell migration^27^. Therefore, we next explored the specific requirement of phosphorylation of PKD2 at Y87 in the adhesion process. Transfection of cells with PKD2 or PKD2.Y87F did not lead to detachment of cells (Supplemental Figure S6A), but cells had significant difficulties to re-adhere to fibronectin-treated coverslips after reseeding when expressing the PKD2.Y87F mutant (Supplemental Figure S6B). Quantification analyses indicated that 4 hours after reseeding, approximately 45% of cells expressing GFP-PKD2 had attached, but when GFP-PKD2.Y87F was expressed this was reduced to about 30%. We noted a similar pattern for the 12 hour time point, for GFP-PKD2 about 80% of cells had attached, but with mutant GFP-PKD2.Y87F this was reduced to approximately 50% (Fig. 6C). Similar negative effects on cell adhesion were obtained when PKD2 was knocked down (Fig. 6D). Since cell adhesion is essential for cell migration, we next compared cells transfected with the Y87F mutant to cells transfected with the wildtype allele of PKD2, and found that the cells expressing the mutant showed a significant decrease in their ability to migrate (Fig. 6E, Supplemental Figure S6C).

Overall, our results show that the RhoA-Src-Y87-PKD2 pathway is important for cell adhesion, and that inhibition of PKD2 phosphorylation at Y87 delays the adhesion process and decreases the ability of cells to migrate.

## Discussion

PKD2 has been implicated in controlling diverse functions within cancer cells, including cell migration^1^, expression and secretion of MMPs and cell invasion^8, 9^, tumor cell proliferation and tumor growth^10, 11^, and chemoresistance^28^. This multitude of different functions can be due to specific binding partners, intracellular location of PKD2 activation or to specific phosphorylation of different motifs. We here focus on such a motif (Y-G-M/L-Y) that is shared by PKD1 and PKD2, but not PKD3 (Fig. 1A)^22^. By using cell lines, which lack expression of PKD1, we specifically were able to investigate the role of phosphorylation of this motif in PKD2. While we previously have shown that in PKD1 the first tyrosine residue (Y95) within the Y-G-M/L-Y motif can be phosphorylated downstream of oxidative stress^22^, we here show that in PKD2 the corresponding tyrosine residue (Y87) is phosphorylated downstream of RhoA and Src (Figs 3, 4 and 5). While in multiple signaling cascades RhoA and Src function synergistically^26^, in our setting, the Src-dependent phosphorylation of PKD2 was downstream of RhoA (Fig. 5).

In order to determine the functional consequence of PKD2 phosphorylation at Y87, we used our anti-pY95/87 antibody that specifically detects phosphorylation at the Y-G-M/L-Y motif^22^. By showing that Y87-phosphorylated PKD2 co-localizes with paxillin a *bona fide* FA protein, and that F-actin filaments terminate at the pY87-PKD2-positive adhesion sites, we identified PKD2 to be located at the focal adhesions (Figs 1, 2 and Supplemental Figure S1C). Additional evidence for PKD2 localization to the FAs is that it co-immunoprecipiates with Paxillin (Supplemental Figure S2A) and other *bona fide* FA proteins such as FAK (Fig. 6A).

FAs are known to be highly tyrosine phosphorylated^29^. Tyrosine phosphorylation has a role in recruitment of proteins to the FAs, but also occurs at the FAs^30, 31^. For example, during the adhesion process, Paxillin is recruited to nascent adhesions and this is accompanied by its phosphorylation at Y31 and Y118^32, 33^. At the FAs, Src seems to have key roles in mediating phosphorylation of FA proteins such as FAK, Paxillin and p130Cas^32, 34, 35^. Here we demonstrate that inhibition of RhoA-Src signaling is associated with reduced detection of pY87-PKD2 at the FAs (Figs 3 and 5).

Tyrosine phosphorylation of PKD2 at Y87 also contributes to its activation, since a PKD2.Y87F mutant was less phosphorylated in its activation loop, and showed less autophosphorylation activity, both direct measures for PKD activity (Fig. 4D,E). Activation loop phosphorylation of PKD usually is mediated by novel Protein Kinase C (nPKC) isoforms. For oxidative stress-induced activation of PKD1, phosphorylation of Y95 in the pY95-G-M-Y motif provides a binding motif for the C2 domain of PKCδ, which leads to activation loop phosphorylation through PKCδ^22^. In contrast, PKD2, after phosphorylation at this motif, did not bind to PKCδ (data not shown), possibly due to the minimal difference in its amino-acid sequence (pY87-G-L-Y). Therefore, we conclude that Y87 phosphorylation is important for PKD2 localization to the FAs, which brings the kinase into proximity to another, yet unidentified, nPKC that mediates activation loop phosphorylation.

PKD2 regulation at Y87 at the FA seems to contribute to cell adhesion (Fig. 6C), as well as cell migration (Fig. 6E). These findings are supported by previous work showing that Src null fibroblasts exhibit defects during the early phases of cell adhesion^36^. In addition, Src can induce adhesion turnover which also contributes to cell migration^21^.

Now that we have identified RhoA-Src-pY87-PKD2 signaling as a mechanism to target PKD2 to the FAs, prospective targets for PKD2 in this pathway still need to be identified in future studies. Possible substrates for PKD2 at the FAs could include FA-localized phosphatidylinositol-4-phosphate 5-kinase type-l γ (PIP5Klγ), which previously has been shown to be a target for PKD1^37^, and depletion of PIP5Klγ leads to severe attachment and cytoskeletal defects in cells^38^. PIP5Klγ regulates PI4,5P_2_ levels, and such alterations regulate proper FA function and FA dynamics^39, 40^. The protrusive forces that regulate cell spreading, as well as dynamic regulation of FAs, are also linked to actin cytoskeletal reorganization^41, 42^. Cofilin/ADF is a key factor for actin re-organization events that is regulated by its phosphorylation state^43, 44^. PKD2 has been shown to regulate the phosphorylation status of cofilin by activating PAK4^45^ and decreasing activity of SSH1L^5, 23^. However, if this occurs at FA and if this is dependent on Y87 phosphorylation remains elusive.

In summary, we identify focal adhesions as previously undescribed sites of PKD2 localization. This localization is characterized by its phosphorylation at Y87, which is mediated by Src downstream of RhoA. Attenuation of this phosphorylation antagonizes cell adhesion and cell migration (Fig. 6F).

## Materials and Methods

### Cell lines, Antibodies and Reagents

Cell lines were obtained from ATTC (Manassas, VA) and cultured in DMEM supplemented with 10% FBS (plus 10 µg/ml insulin for NMuMG). Primary antibodies are described in detail in Supplemental Table 1 and were from Santa Cruz Biotechnology (Dallas, TX), Sigma-Aldrich (St. Louis, MO), BD Transduction Laboratories (Franklin Lakes, NJ), Invitrogen (Carlsbad, CA), Cell Signaling Technology (Danvers, MA), Bethyl Laboratories (Montgomery, TX) and from Millipore (Billerica, MA). The generation of the affinity-purified rabbit polyclonal antibody anti-pY95/87 (PKD1/PKD2) had been described before^22^. Secondary HRP-linked antibodies were from Millipore. Secondary antibodies (Alexa Fluor 546 F(ab’) fragment of goat anti-rabbit IgG (H + L), Alexa Fluor 488 F(ab’)2 fragment of goat-anti-rabbit IgG, Alexa Fluor 568 F(ab’)2 fragment of goat-anti-mouse IgG) were from Invitrogen (Carlsbad, CA). Alexa Fluor 633-phalloidin and Rhodamine-phalloidin were from Invitrogen. Zenon Alexa Flour (rb-488, rb-405 and mAb IgG1–647) labeling kits were from Thermo Fisher Scientific (Waltham, MA). For transient transfection of HeLa cells, TransIT-HeLa Monster (Mirus Bio, Madison, WI) was used. Recombinant human PKD2 (cat # 14–506) and Src (cat # 14–326) were from Millipore. Fibronectin was from Sigma-Aldrich, PP2 was from Millipore, and Rho Inhibitor I was from Cytoskeleton Inc (Denver, CO). All other chemicals were from Thermo Fisher Scientific.

### DNA Expression and Lentiviral shRNA Expression Constructs

The expression plasmids for constitutively-active Src (Src.Y527F), pEBG-RhoA.CA, Rac1.CA, Cdc42.CA and FLAG-tagged PKD2 have been described before^22, 24^. RFP-LifeAct was from ibidi (Martinsried, Germany). GFP-tagged PKD2 was from Dr. T. Seufferlein (Universitätsklinikum Ulm, Germany). PKD2.Y87F mutations were introduced by site-directed mutagenesis (QuikChange Site-Directed Mutagenesis kit, Agilent Technologies, Santa Clara, CA) using 5′-TTCCCTGAGTGTGGCTTCTTCGGCCTTTACGACAAGATC-3′ and 5′-GATCTTGTCGTAAAGGCCGAAGAAGCCACACTCAGGGAA-3′ as primers. Non-targeting (scr) shRNA (SHC001), shRNAs targeting PKD2 (PKD2-shRNA#1, NM_016457.x-1767sc1c1) and PKD2-shRNA#2, NM_016457.x-1335s1c1) as well as shRNAs targeting Src (Src-shRNA#1 NM_198291.1-1579s1c1 and Src-shRNA#2 NM_198291.1-648s1c1) were from Sigma-Aldrich.

### Reverse Transcriptase-Polymerase Chain Reaction (RT-PCR)

Cellular RNA was isolated using RNA-Bee (TEL-TEST, Friendswood, TX) according to the manufacturer’s instructions and mRNA was transcribed into cDNA using Superscript II (Invitrogen). For the transcription reaction 1 µg Oligo dT(18) primer (New England Biolabs, Beverly, MA) and 1 µg RNA were incubated in a total volume of 10 µl H_2_O at 70 °C for 10 min. 5x buffer, 40 U RNAsin (Roche, Mannheim, Germany), 200 µM dNTP (NEB), 10 mM DTT, 300 U Superscript II reverse transcriptase were added to a total volume of 20 µl. The reaction was carried out at 45 °C for 60 minutes and then heat-inactivated at 95 °C for 5 minutes. Resulting cDNA was subjected to PCR analysis using specific primer sets. Primers were: 5′-TTCTTCCACCTCAGGTCATC-3′ and 5′-TGCCAGAGCACATAACGAAG-3′ for a 600 bp fragment of human PKD1, 5′-CAACCCACACTGCTTTGAGA-3′ and 5′-CACACAGCTTCACCTGAGGA-3′ for a 698 bp fragment of PKD2; and 5′-TCATTGACAAACTGCGCTTC-3′ and 5′-GTACATGATCACGCCCACTG-3′ for a 485 bp fragment of PKD3. Reaction conditions for the PCR reactions were: 1 min annealing at 55 °C, 1 min amplification at 72 °C, with 25 cycles.

### Immunoblotting, Immunoprecipitation and SDS-PAGE

Cells were washed 2 times with ice-cold PBS (140 mM NaCl, 2.7 mM KCl, 8 mM Na_2_HPO_4_, 1.5 mM KH_2_PO_4_ pH 7.2) and lysed with Buffer A (50 mM Tris-HCl pH7.4, 1% Triton X-100, 150 mM NaCl, 5 mM EDTA pH 7.4) containing Protease Inhibitor Cocktail (PIC, Sigma-Aldrich). After 30 minutes of incubation on ice, lysates were centrifuged (13,000 rpm, 15 min, 4 °C) and protein concentration was determined. Proteins were immunoprecipitated by incubation with a specific antibody (2 μg) for 1 hour, after which samples were incubated for a further 30 minutes with Protein G Sepharose (GE Healthcare, Piscataway, NJ). Immunecomplexes were washed 3 times with TBS (50 mM Tris-HCl pH 7.4, 150 mM NaCl), and resolved in 20 μl TBS and 2x Laemmli buffer. Samples were subjected to SDS-PAGE, transferred to nitrocellulose membranes and visualized by immunostaining.

### *In Vitro* Kinase Assay

The *in vitro* protein kinase assays were performed by adding 100 ng of active human Src to 100 ng human PKD2 (Millipore) in 40 µl of kinase buffer (50 mM Tris pH 7.4, 10 mM MgCl_2_, 2 mM DTT) supplemented with 100 μM ATP. The reaction was incubated for 30 minutes at room temperature and was stopped by addition of 2x Laemmli buffer.

### Immunofluorescence

Cells were transfected or stimulated and seeded in ibidi 6 channel μ-slides (ibidi) or on coverslips (Figs. 5B, C), as indicated in the Figure Legends. For immunofluorescence analyses, cells were washed twice with PBS and then fixed with 4% paraformaldehyde (15 min, 37 °C). Following fixation, cells were washed 2–5 times with PBS, permeabilized with 0.1% Triton X-100 in PBS (3–5 min, RT) and blocked with 10% NGS, 3% bovine serum albumin and 0.05% Tween 20 in PBS (blocking solution) for 30 min at room temperature. Samples were incubated with indicated primary antibodies (for dilutions see Supplemental Table 1) in blocking solution either for 1 hour at RT or overnight at 4 °C. Following five washes with PBS, samples were incubated (1 hour, RT) with secondary antibodies at 1:800 in blocking solution or Alexa Fluor 633-phalloidin or Rhodamine-phalloidin diluted at 1:200. After further washing with PBS, ibidi mounting media (ibidi) was added to the cells in the channel slide. Cells on coverslips were mounted onto slides using Fluoromount G (Thermo Fisher Scientific) or Lab Vision^TM^ PermaFluor^TM^ Aqueous Mounting Medium (Thermo Fisher Scientific). Samples were evaluated using either a LSM 880 or a LSM 510META confocal laser scanning microscope (Zeiss, Jena, Germany), or an IX81 DSU Spinning Disc Confocal (Olympus, Center Valley, PA).

### Adhesions Assays

Cells were transfected or infected as indicated and then reseeded onto fibronectin-coated glass coverslips (10 μg/ml) at a density of 0.02 × 10^6^ cells/coverslip. Cells were imaged at the indicated times and counted manually to determine the number of cells attached.

### Cell Migration Assays

Transwell migration assays were performed as previously described^3^. Briefly, cells were harvested, washed once with media containing 1% bovine serum albumin (BSA) and re-suspended in media containing 0.1% BSA. Then 100,000 cells were seeded per transwell insert. NIH-3T3 conditioned medium served as a chemoattractant in the lower chamber. Remaining cells were used to control the expression of genes of interest by Western blot. After 16 hours, cells on top of the transwell insert were removed and cells that had migrated to the lower surface of the filters were fixed in 4% paraformaldehyde and counted.

### Statistical Analysis

Data are presented as mean ± SEM. P values were acquired with the student’s *t*-test using Graph Pad software, and p < 0.05 was considered statistically significant.

### Data Availability

All data generated or analyzed during this study are included in this published article (and its Supplementary Information files).

## Electronic supplementary material

Supplementary Data
