# Supplementary material for: Src-mediated tyrosine phosphorylation of Protein Kinase D2 at focal adhesions regulates cell adhesion
Source: Sci Rep. 2017 Aug 25;7:9524. doi: 10.1038/s41598-017-10210-7 (PMC5573332; doi:10.1038/s41598-017-10210-7)
Supplement: Supplementary file 1 — Supplementary Data [file 41598_2017_10210_MOESM1_ESM.pdf]

Supplementary Information
for

Src-mediated tyrosine phosphorylation of Protein Kinase D2 at focal adhesions regulates cell adhesion

Nisha Durand[1], Ligia I. Bastea[1], Heike Döppler[1], Tim Eiseler[2] and Peter Storz[1,3]

[1] Department of Cancer Biology, Mayo Clinic, Jacksonville, Florida 32224, USA
[2] Department of Internal Medicine I, Ulm University, D-89081 Ulm, Germany
[3] Corresponding author: Peter Storz, Mayo Clinic, 4500 San Pablo Road, Jacksonville, FL 32224. Phone: 904 953-6909, FAX: 904 953-0277, e-mail: storz.peter@mayo.edu

Supplemental Figure S1, relates to Figure 1

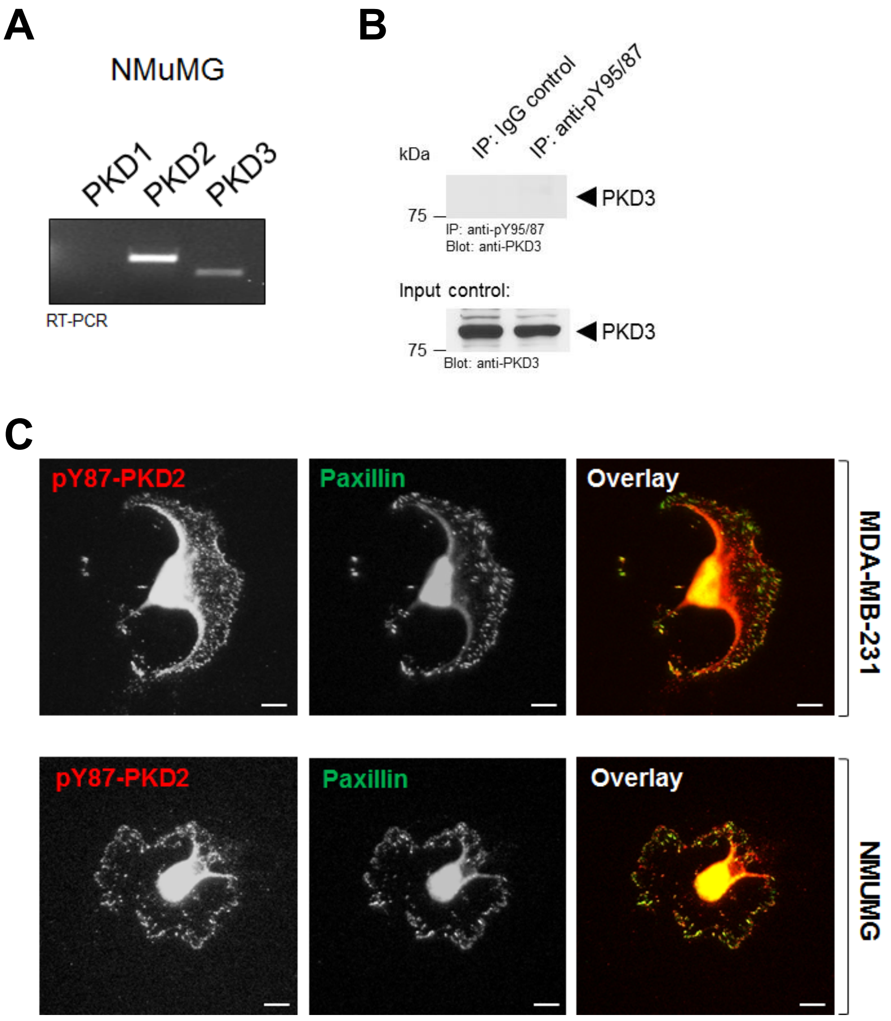

Supplemental Figure S1, relates to Figure 1: **A: NMuMG cell express PKD2 and PKD3, but not PKD1.** RT-PCR analysis using specific primer sets for mouse PKD1 (CTGGACATGTGGTCTGTTGG and GCTCACTGAGGGCTTTCATC), PKD2 (GCAGATATCCAGGAGGGTGA and GCACAGCGTTTGTGACAGTT) and PKD3 (CATCAGCAGCGTTTACCAGA and CACACAGCTTCACCTGAGGA). All experiments have been performed at least three times with similar results. **B: PKD3 can not be immunoprecipitated with the anti-pY95/87 antibody.** HeLa cells (3.5 x 10^6 cells per 10 cm plate) were subjected to immunoprecipitation analysis using the anti-pY95/87 antibody, and then probed for PKD3. Control Western blots of lysates were evaluated for PKD3 expression (input). **C: Y87-phosphorylated PKD2 co-localizes with Paxillin.** MDA-MB-231 and NMuMG cells in ibidi channel µ-slides were analyzed by immunofluorescence to determine the localization of endogenous pY87-PKD2 and Paxillin (secondary antibodies used: Alexa Fluor 488, Alexa Fluor 568). Scale bars indicate 10 µm.

Supplemental Figure S2, relates to Figure 1

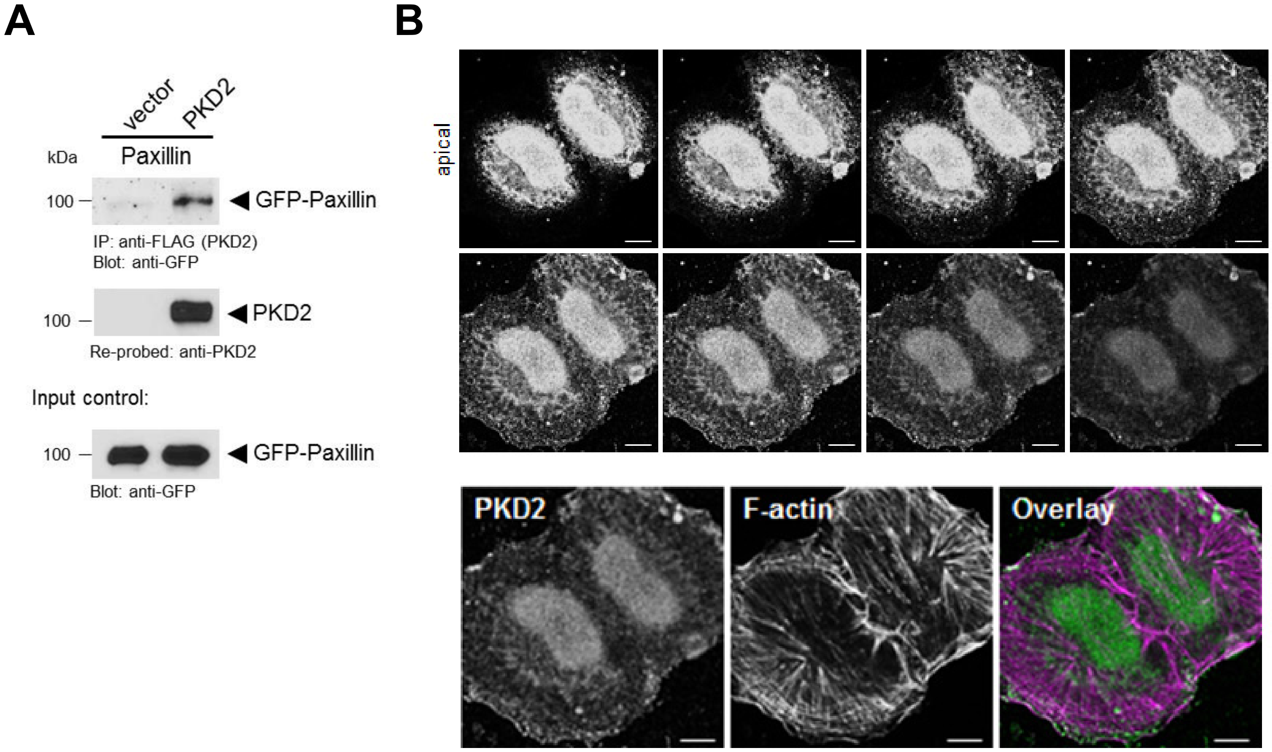

Supplemental Figure S2, relates to Figure 1: **PKD2 can be detected at the focal adhesions. A:** Hek293T cells (0.5 x 10^6 cells/well of a 6 well plate) were co-transfected with GFP-Paxillin and vector or FLAG-PKD2. PKD2 was immunoprecipitated (anti-FLAG) and samples were analyzed for co-immunoprecipitated Paxillin (anti-GFP), and then then re-probed for PKD2. Control Western blots of lysates were evaluated for GFP-Paxillin expression (input) using anti-GFP antibodies. **B:** HeLa cells (6000 cells/channel, ibidi channel µ-Slide) were analyzed for the localization of endogenous PKD2 by immunofluorescence analysis (anti-PKD2 and Alexa Flour 488). F-actin was visualized with Alexa Flour 633-Phalloidin. Images shown represent single Z-stack slices (apical to basal, step size 0.5 µm). Scale bars indicate 10 µm.

Supplemental Figure S3, relates to Figure 2

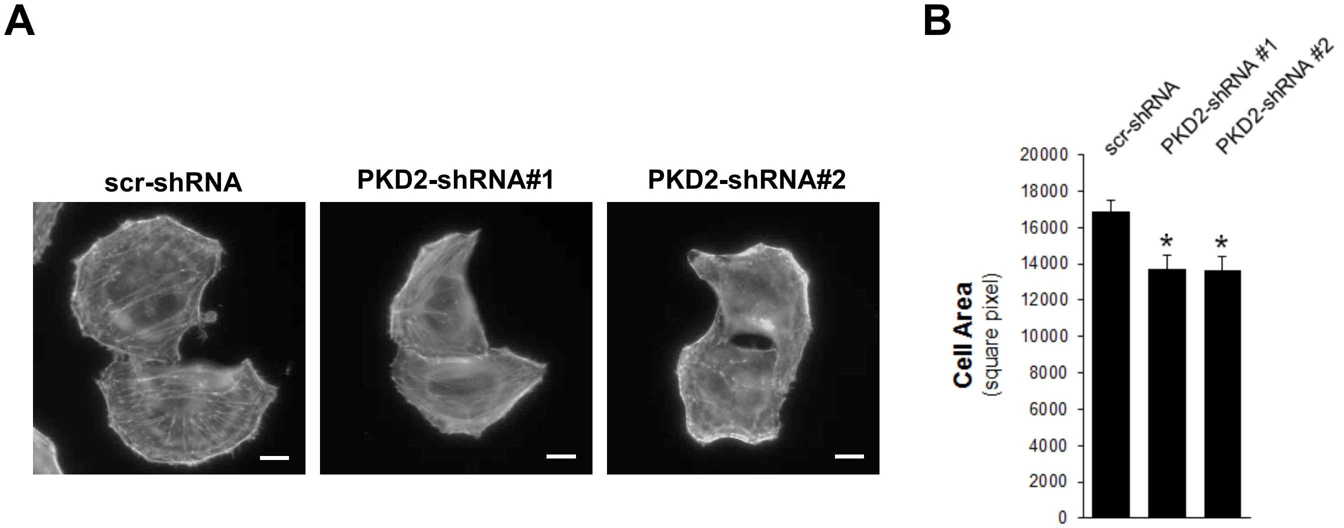

Supplemental Figure S3, relates to Figure 2: **Loss of PKD2 is associated with reduced cell spreading. A:** HeLa cells (0.2 x 10^6 cells/well, 6-well plate) were infected with lentivirus expressing non-target (scr) shRNA, or two independent shRNA sequences targeting PKD2. After 48 hours, cells (6000/channel) were reseeded in ibidi channel slides. Cells were subjected to immunofluorescence analysis to visualize F-actin which was stained with phalloidin. The scale bar indicates 10 µm. **B:** Shows quantitation for cell area, determined using Image J software in cells expressing scr-shRNA (n=50), PKD2-shRNA#1 (n= 50) and PKD2-shRNA#2 (n=50). The asterisk indicates statistical significance ($p < 0.05$) as compared to cells expressing scr-shRNA.

Supplemental Figure S4, relates to Figure 3

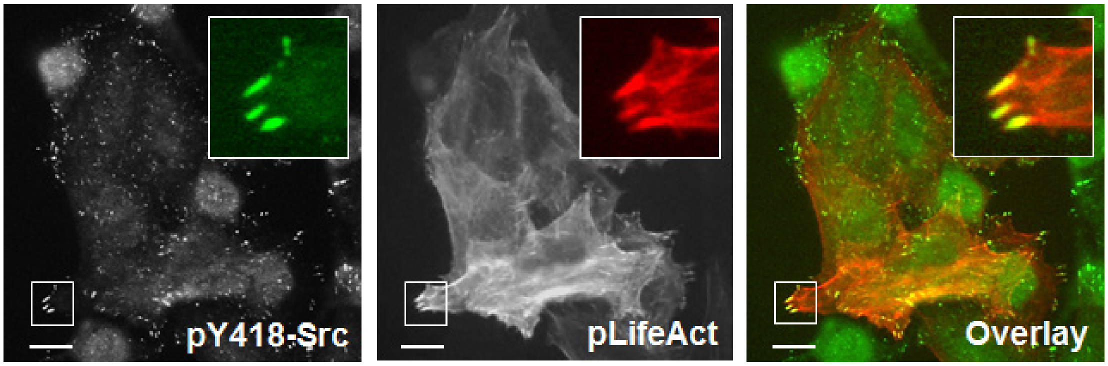

Supplemental Figure S4, relates to Figure 3: **Detection of active Src at focal adhesions of HeLa cells growing in channel slides.** HeLa cells in ibidi channel μ-slides were transfected with RFP-LifeAct for visualization of F-actin. Cells were fixed and the localization of endogenous pY418-Src was determined by immunofluorescence analysis (secondary antibody used: Alexa Fluor 488). Scale bars indicate 10 μm.

Supplemental Figure S5, relates to Figure 5

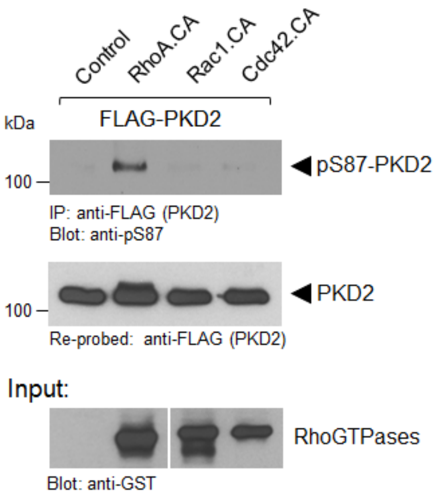

Supplemental Figure S5, relates to Figure 5: **Active RhoA, but not active Rac1 or Cdc42 induce PKD2 phosphorylation at Y87.** HeLa cells (3 x 10^5 cells per well, 6 well plate) were co-transfected with FLAG-tagged PKD2 and GST-tagged versions of indicated RhoGTPases or control. PKD2 was immunoprecipitated (anti-FLAG) and samples were analyzed for PKD2 phosphorylation at Y87 (anti-pY87), and then then re-probed with anti-FLAG (PKD2). Control Western blots of lysates were evaluated for GST expression (input) using anti-GST antibodies.

Supplemental Figure S6, relates to Figure 6

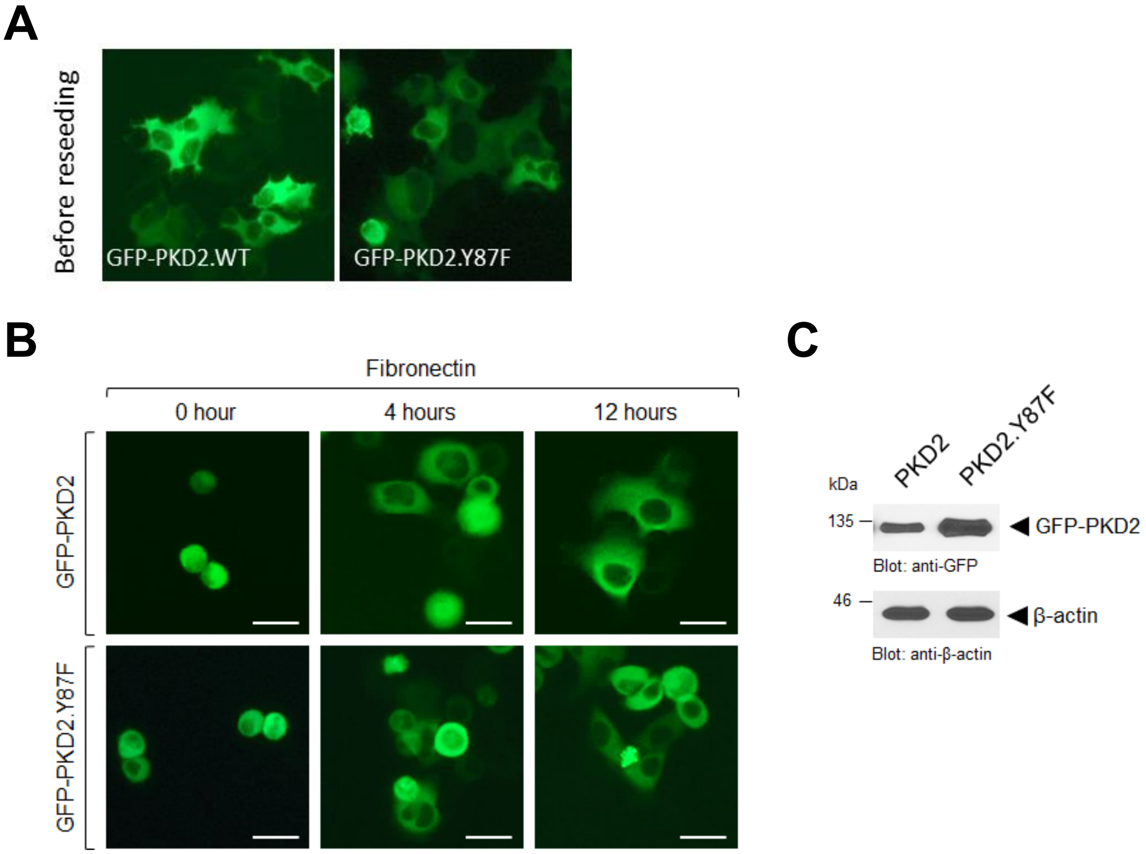

Supplemental Figure S6, relates to Figure 6: **Prevention of Y87-phosphorylation decreases cell adhesion and cell migration. A, B:** HeLa cells were transfected with GFP-tagged versions of PKD2 or PKD2.Y87F (**A** shows cells before reseeding) and then re-plated on fibronectin-coated (10 μg/ml) glass coverslips at a density of 0.02 x 10^6 cells/coverslip. Fluorescent images were acquired at the indicated times to determine the number of cells attached. **B** shows representative areas. Scale bar indicates 25 μm. A quantitation for the percentage of cells attached at 4 and 12 hours is shown in **Figure 6B** of the main manuscript. **C:** Western blots corresponding to **Figure 6E**. Indicated samples were analyzed for expression of GFP-tagged wildtype or mutant PKD2 using anti-GFP antibodies. Staining of the same blots for β-actin (anti-β-actin antibody) served as a loading control.

Supplemental Figure S7, uncropped blots Figures 1C, 2C, 3C and 4A

Figure 1C:

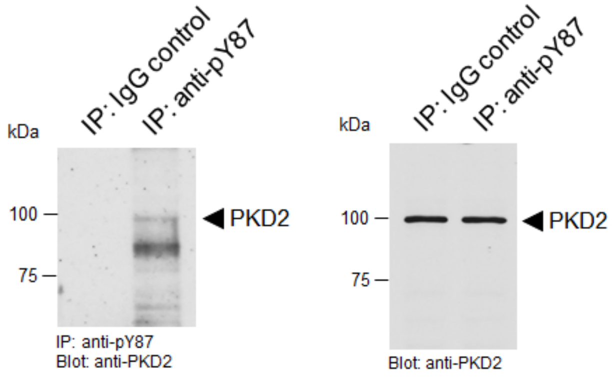

Figure 2C:

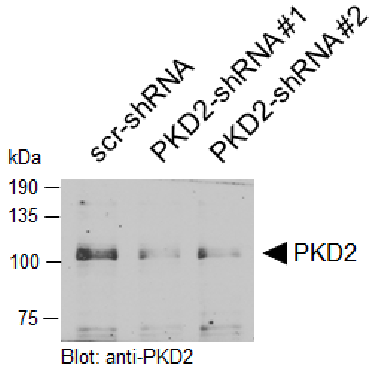

Figure 2C:

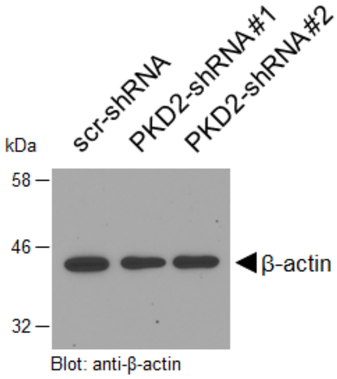

Figure 3C:

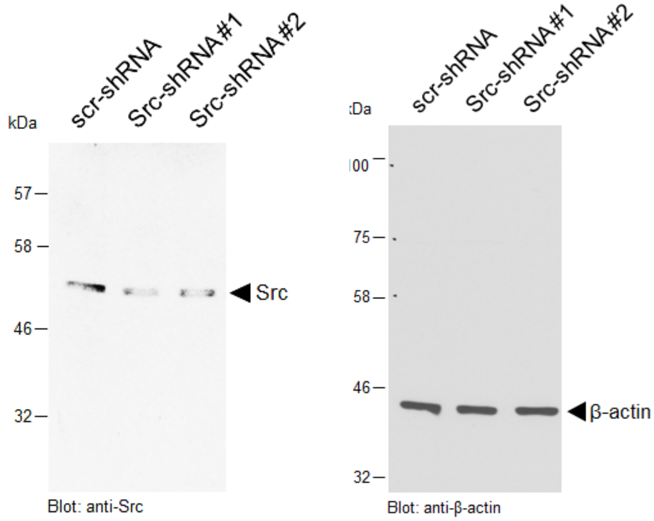

Figure 4A:

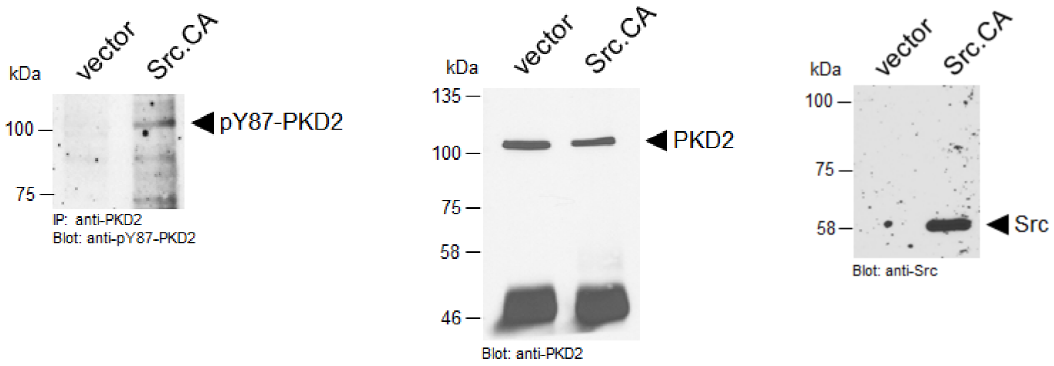

Supplemental Figure S8, uncropped blots Figures 4B, 4C and 4D

Figure 4B:

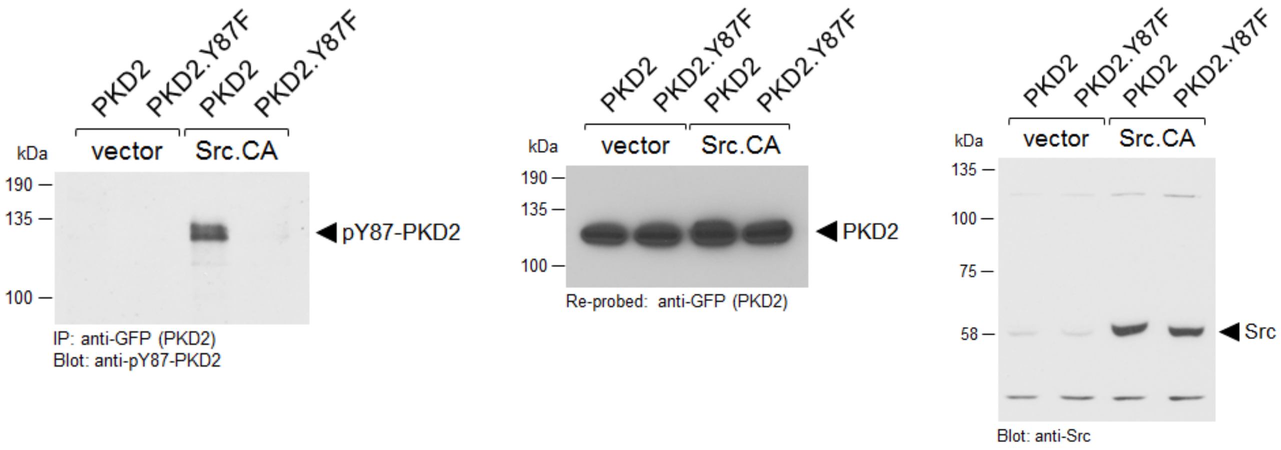

Figure 4C:

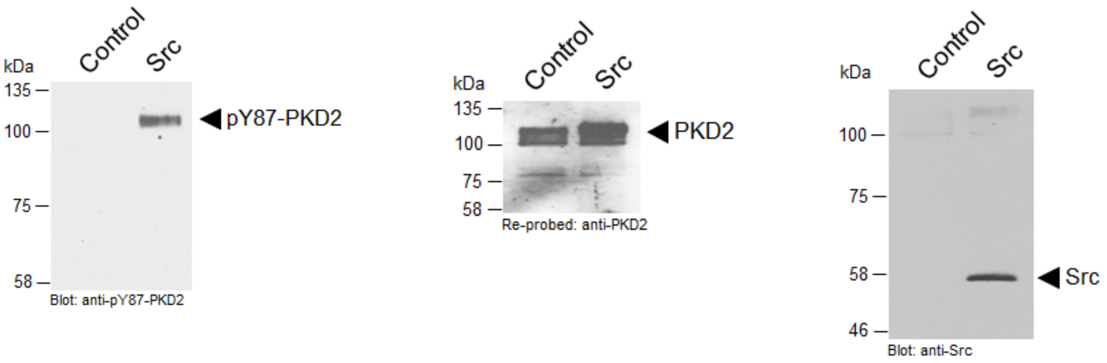

Figure 4D:

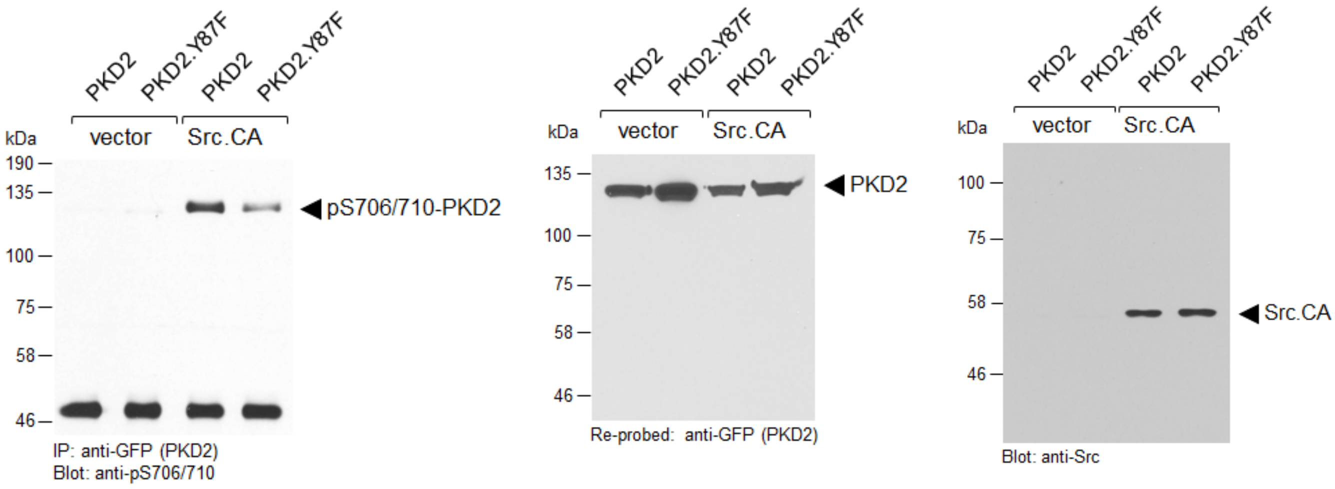

Supplemental Figure S9, uncropped blots Figures 4E, 5A and 6A

<u>Figure 4E:</u>

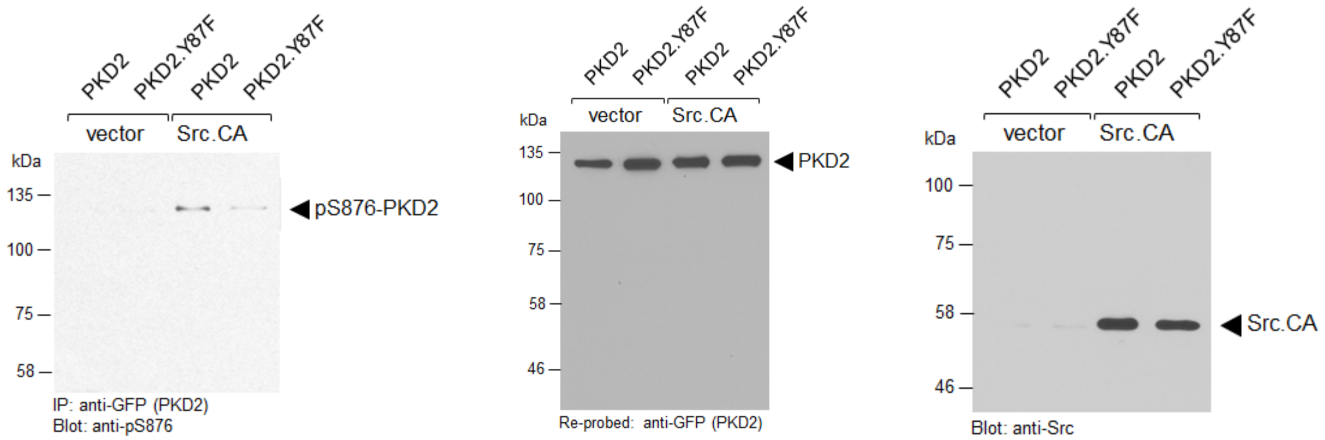

<u>Figure 5A:</u>

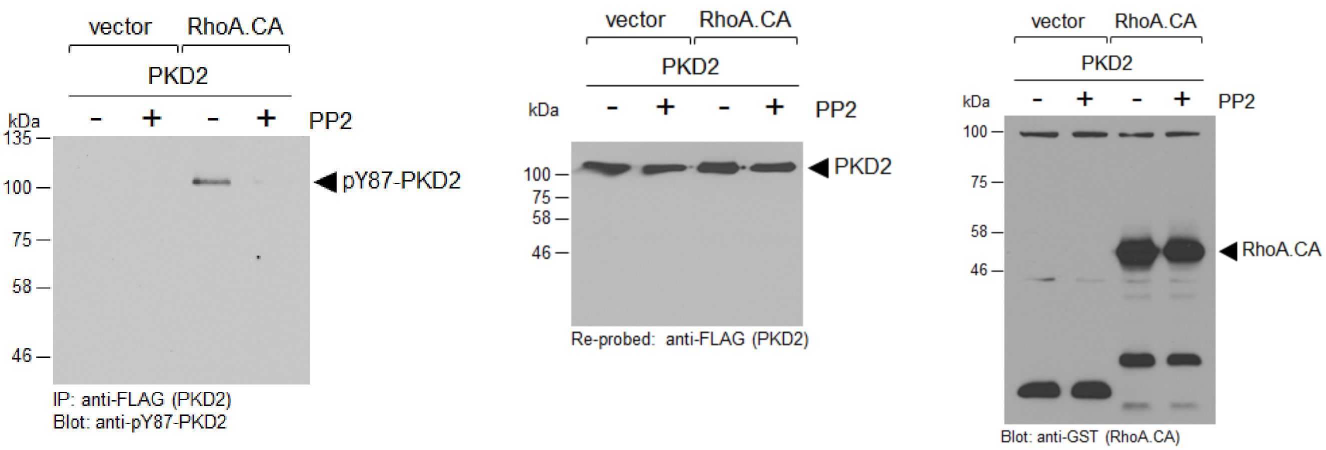

<u>Figure 6A:</u>

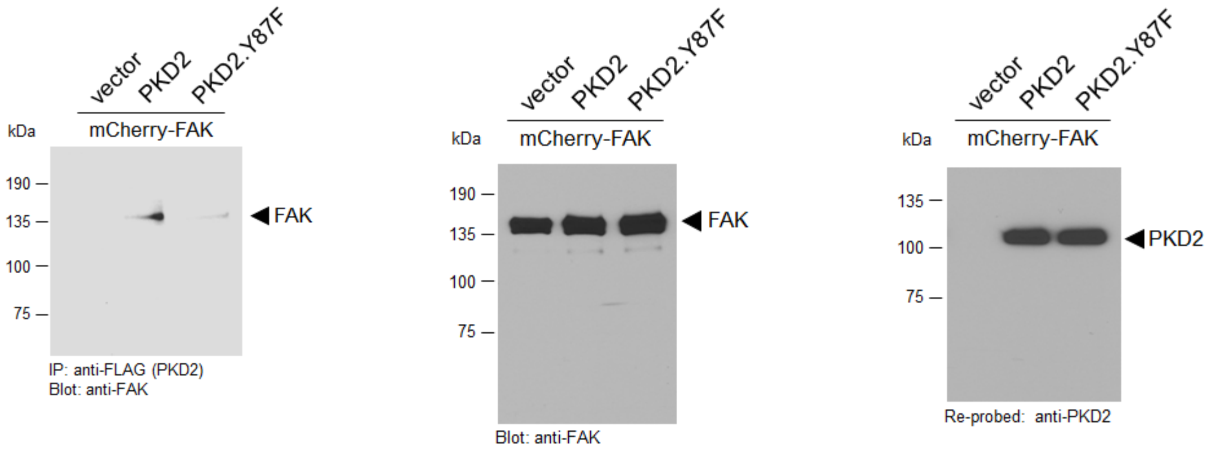

Supplemental Table 1

**Antibody**	**Source**	**Catalog Number**	**IF**	**IP**	**WB/IB**
FAK	BD Transduction Laboratories	610087	1:200		1:1000
FLAG (M2)	Sigma-Aldrich	F3165		1 µl/sample	1:2000
GFP (B2)	Santa Cruz	sc-9996		1.5 µl/sample	1:2000
GST (Z5)	Santa Cruz	sc-459	1:200		1:2000
Paxillin (5H11)	Millipore	05-417	1:300		
PKD1/2 (C-20)	Santa Cruz	sc-639		5 µl/sample	1:2000
PKD2	Bethyl Laboratories	A300-073A			1:1000
PKD2	Abcam	Ab51250	1:250		
PKD3	Bethyl Laboratories	A300-319A2			1:1000
pS744/748 (pS706/710 in PKD2)	Cell Signaling Technology	#2054			1:1000
pS916 (pS876 in PKD2)	Cell Signaling Technology	#2051			1:2000
pY397-FAK	Invitrogen	44-625G	1:300		1:1000
pY418-Src-Alexa 488	Invitrogen	44660A1	1:250		
pY95/87	Storz Laboratory		1:250	10 µl/sample	1:500
Src	Santa Cruz	SC-18			1:200
Src (GD11)	Millipore	05-184			1:1000
Vinculin	Sigma-Aldrich	V4505	1:300		1:1000
β-actin	Sigma-Aldrich	A5441			1:10000

Supplemental Table 1, relates to Materials & Methods, section: **Cell lines, Antibodies and Reagents**. Listed are all antibodies used including their source, catalog number and use in immunofluorescence (IF), immunoprecipitation (IP) and Western- or Immunoblotting (WB/IB).
